# Mouse *Tbx3* Mutants Suggest Novel Molecular Mechanisms for Ulnar-Mammary Syndrome

**DOI:** 10.1371/journal.pone.0067841

**Published:** 2013-07-02

**Authors:** Deborah U. Frank, Uchenna Emechebe, Kirk R. Thomas, Anne M. Moon

**Affiliations:** 1 Department of Pediatrics, University of Utah, Salt Lake City, Utah, United States of America; 2 Department of Neurobiology and Anatomy, University of Utah, Salt Lake City, Utah, United States of America; 3 Department of Human Genetics, University of Utah, Salt Lake City, Utah, United States of America; 4 Molecular Medicine Program, University of Utah, Salt Lake City, Utah, United States of America; 5 Weis Center for Research, Danville, Pennsylvania, United States of America; Northwestern University, United States of America

## Abstract

The transcription factor TBX3 plays critical roles in development and *TBX3* mutations in humans cause Ulnar-mammary syndrome. Efforts to understand how altered TBX3 dosage and function disrupt the development of numerous structures have been hampered by embryonic lethality of mice bearing presumed null alleles. We generated a novel conditional null allele of *Tbx3:* after Cre-mediated recombination, no mRNA or protein is detectable. In contrast, a putative null allele in which exons 1-3 are deleted produces a truncated protein that is abnormally located in the cytoplasm. Heterozygotes and homozygotes for this allele have different phenotypes than their counterparts bearing a true null allele. Our observations with these alleles in mice, and the different types of *TBX3* mutations observed in human ulnar-mammary syndrome, suggest that not all mutations observed in humans generate functionally null alleles. The possibility that mechanisms in addition to *TBX3* haploinsufficiency may cause UMS or other malformations merits investigation in the human UMS population.

## Introduction

The transcription factor TBX3 is critical for human development: heterozygotes bearing point, deletion and insertion mutations in *TBX3* have ulnar-mammary syndrome (UMS) consisting of congenital limb defects, apocrine and mammary gland hypoplasia, and dental and genital abnormalities [1]. More recently, heart and conduction system defects have been described in mice (*Tbx3*) and humans (*TBX3)* [2], [3], [4], [5], [6], [7]. Abnormal *TBX3* expression occurs in multiple cancers [8]. It is becoming apparent that T-box proteins have functions in addition to transcriptional regulation [9] and that their activities are highly dosage sensitive [5].

We generated novel *Tbx3* gene targeted alleles in mice and found that ablation of different regions of the N-terminal genomic sequence has different molecular and phenotypic consequences. We show that deletion of the T-box encoding region does not inevitably generate a null allele, as has been presumed [2], [3], [4]: splicing of residual 5′ untranslated and 3′ coding sequences can generate an abnormal mRNA that is translated into an aberrant protein predominantly localized in the cytoplasm. Homozygotes for this allele have different phenotypes than those observed with a true null allele that produces no mRNA or protein. These findings have important implications for interpreting the phenotypes of other presumed null alleles of *Tbx3* in mice, and when considering the molecular mechanisms of congenital defects in humans with different types of *TBX3* mutations..

## Results

Three gene targeted *Tbx3* alleles have been previously reported by other laboratories. These alleles were presumed null because they either delete exons encoding the T-box and DNA binding domain (*Tbx3^tm1Pa^* [2]), or have insertions that disrupt the normal translational start codon *Tbx3^Cre^* [3] and *Tbx3^neo^* [4]. Homozygotes for these *Tbx3* alleles die in broad windows from embryonic day (e) 10.5-e16.5. Heterozygotes are reportedly normal with the exception of mildly abnormal external genitalia in the *Tbx3^tm1Pa/tm1Pa^* mutant females [2]. The bases of the variable phenotypes observed with these alleles have not been determined: different genetic backgrounds likely play a role however, the possibility that these mutant alleles produce aberrant forms of Tbx3 mRNA and protein has not been tested.

**Figure 1:**
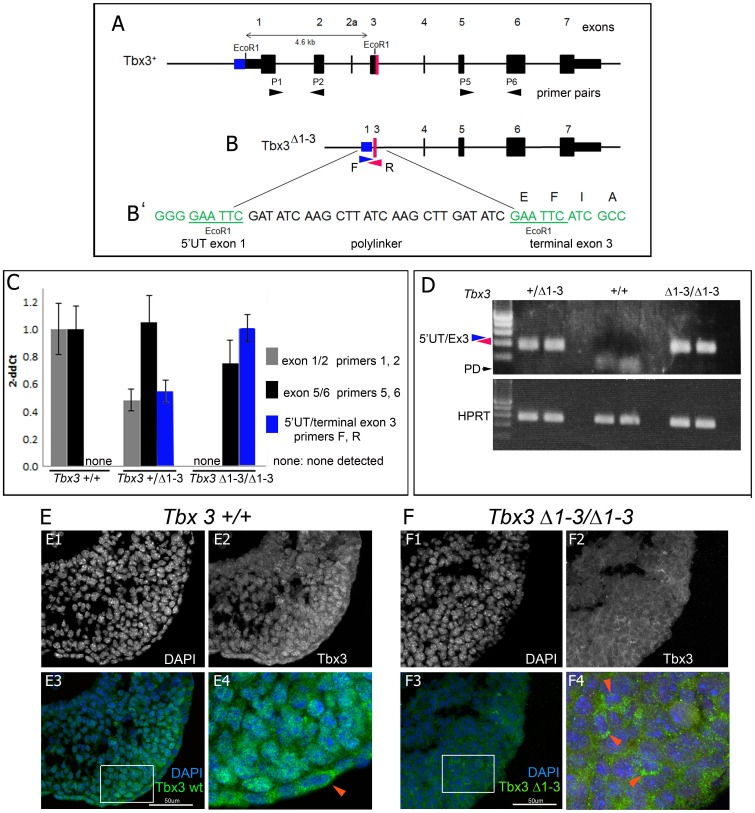
Deletion of the N-terminal region of the *Tbx3* locus results in production of an abnormal protein. A) Schematic of wild type mouse *Tbx3* locus. Exons are depicted in thick black bars and numbered 1–7. Black arrowheads beneath locus indicate location of primers used for PCR analyses in panels C and D. Bidirectional arrow above wild type locus indicates region deleted between two endogenous EcoRI sites to generate the *Tbx3^Δ1-3^* targeted allele shown in B. Blue and red regions indicate portions of Exon1/5′UTR and exon 3 that remain post targeted deletion. B) The *Tbx3^Δ1-3^* allele. Blue and red regions indicate portions of Exon1/5′UTR and exon 3 that remain post targeted deletion. The red and blue arrowheads indicate primers (F, R) used for PCR analyses to assay for novel mRNA spanning the deletion. B’) Genomic sequence resulting from deletion between endogenous EcoRI sites and insertion of polylinker to create the *Tbx3^Δ1-3^* allele. Note preservation of reading frame from exon 1 through polylinker into exon 3, which would produce a protein with wild type sequence beginning in exon 3. C) Quantitative real-time PCR analysis of cDNA from reverse transcribed *Tbx3* mRNA in e10.5 mouse embryos. Bar graphs compare levels of transcripts detected using primer sets depicted in panels A and B. In *Tbx3^+/+^* embryos, no transcripts reflecting splicing from the 5′UTR into exon 3 are detected (none). In *Tbx3^+/Δ1-3^* heterozygotes, 50% of wild type levels of exon 1/2 containing transcripts are present as expected from the single wild type allele. 100% of wild type levels of exon 5/6 containing transcripts are present (black bar). This reflects the contributions from the wild type allele (gray bar) and from the abnormal mRNA containing C-terminal sequences as detected with the F/R primer set (blue bar). In *Tbx3^Δ1-3/Δ1-3^* homozygotes, no exon 1/2 containing transcripts are present (none) and aberrant transcripts from the *Δ*1-3 allele (blue bar) are present at levels comparable to that of wild type transcripts in wild type embryos. D) Visualization PCR products of reverse transcribed *Tbx3* mRNA. Amplicons obtained using the F/R primer set shown in panel A, and with primers to detect HPRT as a control, were run on an agarose gel and visualized with ethidium bromide. *Tbx3 Δ1-3* transcripts are not detected in wild type embryos. PD, primer dimer. E, F) The *Tbx^Δ1-3^* mRNA is translated into protein that is predominantly localized to the cytoplasm. Confocal micrographs of sectioned E10.0 limb buds after fluorescent immunohistochemical detection of Tbx3 using an antibody to the C-terminus. E1-4) *Tbx3^+/+^* limb bud. E1-2) DAPI and FITC channels showing DNA and Tbx3 immunoreactivity respectively. E3) Merged color view of E1 and E2. E4) Close up of white boxed region in E3 showing Tbx3 immunoreactivity in nucleus of limb mesenchymal cells and cytoplasm of ectodermal cells (red arrowhead). F1-4) *Tbx3^Δ1-3/Δ1-3^* limb bud. F1-2) DAPI and FITC channels showing DNA and Tbx3 immunoreactivity respectively. F3) Merged color view of E1 and E2. F4) Close up of white boxed region in F3 showing abnormal Tbx3 immunoreactivity in cytoplasm of limb mesenchymal cells (red arrowheads).

We generated a *Tbx3* targeted allele deleting most T-box encoding sequences (*Tbx3^Δ1-3^*, Figure 1A [5]). The coding portions of exons 1, 2, 2a and those encoding the 5′ 37 amino acids of exon 3 were deleted. *Tbx3^Δ1-3/Δ1-3^* homozygotes were rarely recovered in the fetal period: 25% of *Tbx3^Δ1-3/Δ1-3^* mutants were dead by e10.5 and 95% were dead by e12.5 (Table 1). Both sexes of *Tbx3^+/Δ1-3^* mice had reduced fertility and most mothers were poor nurturers. 20% of *Tbx3^+/Δ1-3^* females had imperforate vaginas; this was never observed in wild type littermates. These phenotypes prompted further investigation into the activity of the *Tbx3^Δ1-3^* allele.

**Table 1:** Comparing embryonic lethality and adult phenotypes of Tbx3*^Δ^* ^1-3^ versus Tbx3*^Δ^* ^flox^ bearing mice.

Embryonicsurvival	Tbx3	Tbx3^+/Δ1-3^	Tbx3^Δ1-3/Δ1-3^	Tbx3	Tbx3^+/Δflox^	Tbx3^Δflox/Δflox^
E9.5	20 (23)	48 (46)	24 (23)	16 (19)	38 (38)	21 (19)
E10.5	9 (8)	18 (16)	4 (8)	10 (10)	22(21)	8 (10)
E12.5	15 (11)	28 (22)	**1 (11)#**	8 (9)	22 (17)	4 (9)*#
E13.5	ND	ND	ND	9 (8)	21 (17)	3 (8)*#
E15.5	6 (5)	13 (10)	**1 (5)#**	ND	ND	ND
**Adult phenotypes**						
Infertile female	0/>80	20/38 @	NA	0/12	0/28 @1	NA
Imperforate vagina	0/>80	7/38 @	NA	0/12	0/28 @2	NA
Female Poor nurturing	<5/>80	30/38 @	NA	0/12	0/28 @1	NA

Numbers are shown as: observed (expected). * Arrhythmias as previously reported. # Significantly different from predicted genotype ratios by Pearson’s Chi Square test. @ Significantly different from Tbx3*^+/+^* by Fisher’s two tailed exact test; p<0.0001. @1 Significantly different from Tbx3*^+/Δ1-3^* by Fisher’s two tailed exact test; p<0.0001. @2 Significantly different from Tbx3*^+/Δ1-3^* by Fisher’s two tailed exact test; p<0.01.

We examined the genomic targeted sequence of *Tbx3^Δ1-3^* and found that it has the potential to encode a transcript restoring the normal *Tbx3* reading frame in the terminal portion of Exon 3 (Figure 1B). We used primers to assay *Tbx3* transcripts containing the exon 1/2 junction by qRT-PCR of *Tbx3^+/Δ1-3^* embryos and, as expected, detected 50% of the wild type transcript level. However, primers that assay the exon 5/6 junction (Figure 1C) detected 100% of the wild type transcript level in *Tbx3^+/Δ1-3^* mutants. We then designed primers to assay for the presence of an aberrant transcript that would be the product of the residual 5′UT and exon 3, as predicted in Figure 1B. We detected this transcript by qRT-PCR, and by standard PCR followed by visualization of an amplicon of the predicted size only in embryos bearing the *Tbx3^Δ1-3^* allele. These findings revealed that an mRNA is produced from the remaining C-terminal exons 3–7 of the *Tbx3^Δ1-3^* allele. More importantly, when we employed a custom antibody to a C-terminal peptide unique to Tbx3, we detected Tbx3 protein in wild type embryos as expected (Figure 1E), but also in *Tbx3^Δ1-3/Δ1-3^* embryos (Figure 1F). Tbx3 protein is normally detected predominantly in the nucleus with variable amounts detected in the cytoplasm, depending on cell type. For example in the limb bud, mesenchymal cells contain mostly nuclear Tbx3 protein (Figure 1E4) while ectodermal cells have cytoplasmic protein (Figure 1E4). In contrast, the *Tbx3^Δ1-3^* mutant protein is cytoplasmic, even in mesenchymal cells (Figure 1F4). This is consistent with deletion of a previously described nuclear localization signal [10].

**Figure 2:**
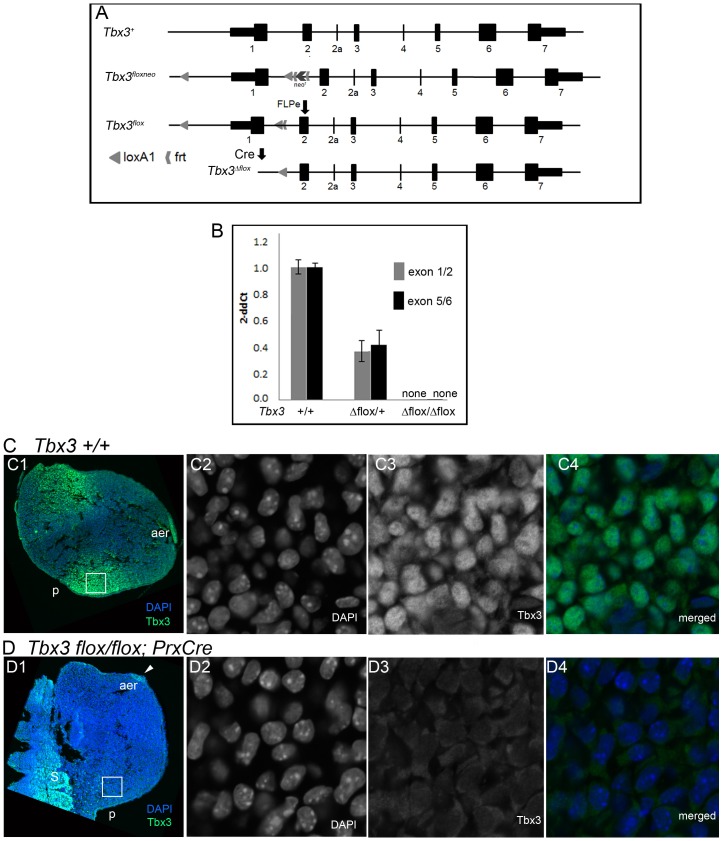
Creation of a *Tbx3* conditional null allele. A) Schematics of wild type mouse *Tbx3* locus and gene targeted alleles. LoxP sites were inserted 3.3 kb 5′ of the translational start and in intron 1 along with an Frt-flanked neomycin resistance selection cassette to create the *Tbx3^floxneo^* allele. FLPe was used to remove the neomycin resistance selection cassette from animals bearing the *Tbx3^floxneo^* allele to generate the *Tbx3^flox^* allele; Cre-mediated recombination generates the *Tbx3^Δflox^* allele. B) Quantitative real-time PCR analysis of cDNA from reverse transcribed *Tbx3* mRNA in e10.5 mouse embryos. Bar graphs compare levels of transcripts detected using primer sets depicted in Figure 1A. In *Tbx3^Δflox/+^* heterozygotes, 50% of wild type levels of exon 1/2 (gray bar) and 5/6 (black bar) containing transcripts are present as expected from the single wild type allele. In *Tbx3^Δflox/Δflox^* homozygotes, no exon 1/2 or 5/6 containing transcripts are present (none). C,D) Confocal micrographs of sectioned E10.0 limb buds after fluorescent immunohistochemical detection of Tbx3 using an antibody to the C-terminus. C1-4) *Tbx3^+/+^* limb bud. C1) Merged color view of DAPI and FITC channels at low magnification. C2-4) Close up of white boxed region in C1. C2) DAPI channel showing DNA immunoreactivity. C3) FITC channel showing Tbx3 immunoreactivity. C4) Merged color view. D1-4) *Tbx3^Δflox/Δflox^*; *PrxCre* limb bud. D1) Merged color view of DAPI and FITC channels at low magnification. D2-4) Close up of white boxed region in C1. D2) DAPI channel showing DNA immunoreactivity. D3) FITC channel showing lack of Tbx3 immunoreactivity. D4) Merged color view.

The need for a *Tbx3* conditional allele that is a true null in the recombined state is clear: it will allow us to bypass embryonic lethality seen in homozygotes of all previously reported mutant alleles (null or otherwise); it will permit conditional gene ablation approaches to study the role of *Tbx3* in specific tissues, at later stages of development and in adult animals; it circumvents hypomorphic phenotypes resulting from insertion of exogenous sequences at other regions of the *Tbx3* locus ([5]; Moon, unpublished). The *Tbx3^flox^* allele (Figure 2A) was designed such that activity of Cre recombinase deletes 4.6kb of genomic DNA encompassing the promoter, 5′UT, transcriptional start site and first exon of *Tbx3* (*Tbx3^Δ^* ^flox^). This design required insertion of two loxP elements at the *Tbx3* locus: one located 3.3 kb 5′ of the translation initiating ATG and the second within the first intron. In addition to mutagenic capacity, site selection was influenced by the absence of any immediately proximate evolutionarily conserved sequences, reducing the chance that the presence of loxP elements would alter gene regulation in *cis*.

The value of *Tbx3^flox^* as a conditional allele requires that it fulfill three requirements: it must behave as wild type in the unrecombined state; it must be a null allele in the recombined state (*Tbx3^Δflox^*); finally, it must be reliably competent for tissue-specific recombination by Cre recombinase. We addressed each of these requirements.

Heterozygous *Tbx3^+/flox^* and homozygous *Tbx3^flox/flox^* mice were indistinguishable anatomically and behaviorally from their wild type siblings, confirming the neutrality of the loxP sites. We generated *Tbx3^+/Δflox^*heterozygotes and unlike *Tbx3^+/Δ1-3^* animals, both sexes were fertile and mothers had normal ability to nurture their litters (N = 28 females, 7 males). Furthermore, no abnormalities of the genitalia were detected.

**Figure 3:**
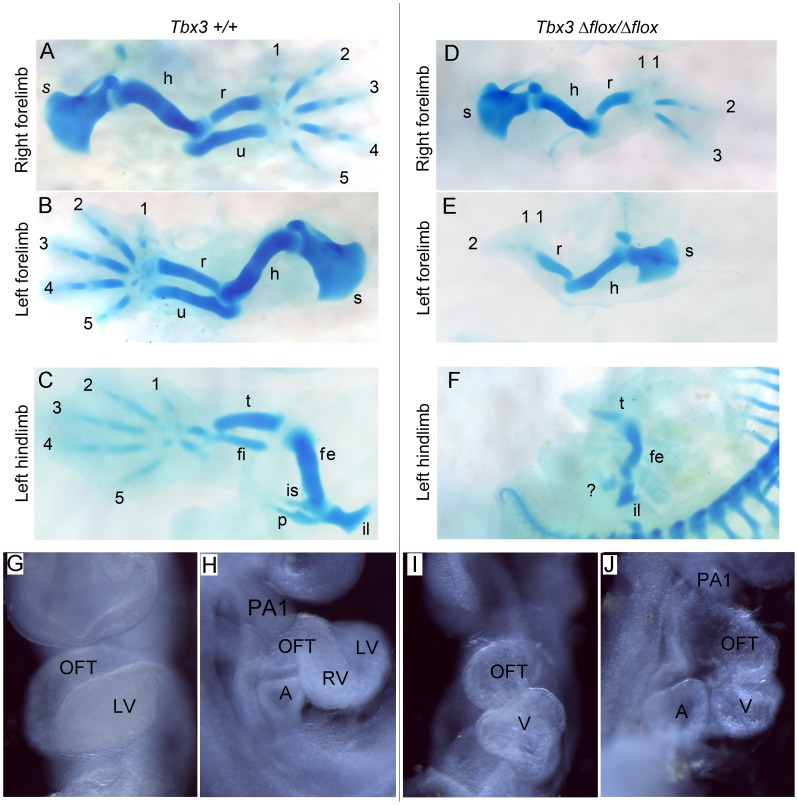
*Tbx3* *^Δ^* ^*flox/Δflox*^ mutants have severe limb and structural heart defects. A–F) Alcian blue treated skeleton preparations of e13.5 control and rare surviving *Tbx3^Δflox/Δflox^* mutants. A–C) Control limbs. D, E) Mutant forelimbs with duplication of digit 1 condensation and loss of digits 4–5 (D, right) or 3–5 (E, left). F) Mutant hindlimbs are truncated beyond the tibia and there is no fibula. The pubis is malformed and lacking one element, likely the pubic bone. s, scapula; h, humerus; r, radius; u, ulna; digits 1–5; fe, femur; fi, fibula; t, tibia; il, ilium; is, ischium; p, pubic bone. G-J) Ventral (G, I) and left lateral (H,J) views of whole mount E9.0 control (G, H) and mutant (I, J) embryos centered on the heart. The mutant heart is short, bowed ventrally rather than the normal loop to the right. Only a single, thin walled ventricular chamber (V) is present whereas in the lateral view of the control, the nascent right ventricle (RV) is present.

In contrast to functional mRNA produced in *Tbx3^Δ1-3/Δ1-3^* embryos, *Tbx3^Δflox/Δflox^* embryos contained no *Tbx3* mRNA when assayed with primers to detect either the 5′ or 3′ ends of the message (Figure 2B). Also in contrast to *Tbx3^Δ1-3/Δ1-3^* embryos (95% which were dead by e12.5), 30% of *Tbx3^Δflox/Δflox^* embryos survived to e13.5 (Table 1). Despite their longer survival, it is notable that the limb and structural heart phenotypes of *Tbx3^Δflox/Δflox^* mutants are more severe than those reported for other presumed null mutants (Figure 3). In E13.5 *Tbx3^Δflox/Δflox^* mutants, we observe truncation of the hindlimbs beyond the tibia, absence of the fibula and an abnormal pelvis in 100% of mutants (N = 6, Figure 3H). In the forelimbs, there is some variability in severity of digit loss such that either digits 4 and 5 or digits 3–5 are absent and the left side is usually more severely affected (Figure 3D,E). There is duplication of the condensations and soft tissue of the first digit. *Tbx3^Δflox/Δflox^* mutant survivor have heart defects similar to those reported in Mesbah et al. [7], although the genetic backgrounds are not the same which makes objective comparison difficult. However, 18/25 *Tbx3^tm1Pa/tm1Pa^* mutants had anterior displacement of the atria and 4/25 had more severe looping defects with thin walled ventricles; most survived to e13.5 with malformed outflow tracts [7]. In contrast, 50% our conditional null homozygotes have severe early looping defects with thin heart tubes and only 3 survived to e13.5; most died prior to the initial stages in outflow tract remodeling. At E9.0 we noted that 50% of *Tbx3^Δflox/Δflox^* mutants have hypoplastic, thin-walled abnormally looped heart tubes (Figure 3 I, J); this phenotype is more severe than reported in *Tbx3^tm1Pa/tm1Pa^* [7], *Tbx3^Cre/Cre^* [3] or *Tbx3^neo/neo^* [4] homozygotes. It would be ideal to examine other presumed null alleles for production of aberrant mRNA/protein and to perform side-by side phenotypic comparison in the same genetic background.

We tested the *Tbx3^flox^* allele for efficient tissue-specific recombination in developing embryos using *Prx1Cre* [11] to recombine the allele in the forelimb mesenchyme. No mesenchymal Tbx3 protein is detectable In *Tbx3^flox/flox^;Prx1Cre* mutants (Figure 2D1, D4), whereas there is robust signal in the control littermate in this tissue (Figure 2C1, C4). Note also that as predicted with *Prx1Cre*, *Tbx3* function and protein production is preserved in the apical ectodermal ridge of the limb bud (Figure 2D1).

## Discussion

UMS phenotypes vary within and between families, and all families evaluated to date have different mutations [6], [12], [13], [14]. This variability, and the absence of malformations in several structures/organs that express *TBX3* during development, suggest that dosage sensitivity to TBX3 is present in human embryonic development as it is in mice: normal murine cardiac structure and function require tight regulation of the dosage of several *Tbx* genes [5], [15], [16], [17].

It has been hypothesized that the mechanism of UMS is *TBX3* haploinsufficiency: transcripts from C-terminal deletion or missense mutant alleles were thought to be functionally null due to degradation by nonsense mediated decay or loss of critical functional domains [12], [14]. Carlson and colleagues subsequently demonstrated that C-terminal truncated proteins could be produced *in vitro* and that a dominant repressor domain in the C-terminus is required for TBX3 to immortalize primary fibroblasts [10]. A correlation between mutations upstream of, or in the T-domain and more severe UMS phenotypes has been suggested more recently [6]. We have now shown that removal of the T-box encoding region allows production of a variant transcript and the resulting protein is aberrantly localized to the cytoplasm. Our findings indicate that the genital, fertility and nurturing phenotypes seen in *Tbx3^+/Δ1-3^* heterozyogotes are not attributable to loss of wild type Tbx3 protein, but due to negative effects of the aberrant protein produced from the *Tbx3^Δ1-3^* transcript. The fact that *Tbx3^Δ1-3/Δ1-3^* homozygotes die earlier than *Tbx3^Δflox/Δflox^* null mutants indicates that deleterious effects of the *Tbx3^Δ1-3^* protein exacerbate those due to of loss of the wild type protein. A similar situation occurs in mice with respect to the *T* gene. Defects in the *Brachyury* mutant (a 200-kb deletion removes the entire gene) are thought to result from haploinsufficiency of the T protein [18]. Other *T* mutations (*T^wis^, T^c^,T^c-2H^*) which encode frameshifts and truncated proteins are thought to generate dominant-negative proteins and cause more severe developmental defects [19]. The heart and skeletal phenotypes of *Tbx3^Δflox/Δflox^* true null mutants are more severe than those of other presumed null mutants previously reported.

Many human UMS mutations have the potential to generate abnormal proteins containing either N- or C- terminal regions. Our observations raise the possibility that some nonsense and missense human *TBX3* mutations previously thought to function as null alleles may also produce aberrant transcripts and proteins with unexpected activity. Since it has been shown that Tbx3 protein has both activator and repressor domains [10], it is likely that a mutant protein with preservation of one domain in the absence of another will have markedly different activity *in* *vivo*.

## Materials and Methods

### Gene Targeting

The *Tbx3^Δ1-3^* allele has a 4.6kb deletion between 2 EcoRI sites located 920 bp 5' of the translational start site and 3.7 kb 3′ of the ATG (Figure 1A). This deletes the a portion of the 5′UTR, all coding regions of exons1, 2 and 2a and all but the terminal portion of exon 3 encoding the final 12 amino acids of this exon. An FRT-flanked neomycin (neo^r^) selection cassette at the BglII site 3.2 kb 5′ of the ATG was removed with Flip recombinase with the B6.SJL-Tg(ACTFLPe)9205Dym/J strain. These mice were maintained in a mixed Bl6/SV129/FVB background.

The *Tbx3^flox^* conditional allele was generated by inserting the 5′ loxP sequence in an AgeI site 4.2 kb upstream of the ATG; the 3′ LoxP sequence was inserted in a SpeI site 1100 bp 3′ of the ATG (midway between the first and second exons, Figure 2A). Adjacent to the 3′ loxP is an FRT-flanked neo^r^ cassette used as a positive selectable marker; a thymidine kinase negative selectable marker was included outside the region of genomic homology. Following electroporation ES cells were selected for G418^r^, Ganc^r^, and 182 cells lines were isolated for further characterization. The initial allele was designated *Tbx3^floxneo^* and ES cells carrying *Tbx3^floxne^* ^o^ were injected into blastocysts to generate chimeric mice that successfully transmitted the mutant allele. Heterozygous *Tbx3^+/floxneo^* progeny of the chimeras were bred with mice expressing FLPe recombinase (*Gt(ROSA)26Sor^tm1(FLP1)Dym^* ) to remove the neo^r^ cassette, creating the *Tbx3^flox^* allele. These mice were maintained in a mixed Bl6/SV129 background.

*Tbx3^+/Δflox^* animals were generated by breeding *Tbx3^flox/flox^* males to *hprtCre* females which causes recombination in the egg; *Tbx3^+/Δflox^* males and females were then intercrossed to obtain.

Tbx3^Δflox/Δflox^ embryos.

### Ethics Statement

All mouse work was performed under a protocol in Dr. Moon’s name approved by the University of Utah IACUC and euthanasia was performed in accordance with AVMA requirements.

### Preparation of RNA from Embryos for Reverse Transcription and qRT-PCR

Tissues were dissected in ice cold PBS and stored in RLT buffer (Qiagen) at −80°C. Total RNA was extracted from samples (RNeasy Micro Kit, Qiagen).One hundred micrograms of total RNA was transcribed to cDNA using the Superscript III First-Strand Synthesis System (Invitrogen). Quantitative PCR was performed with iQ SYBR Green Supermix on the iCycler system (Bio-Rad) and normalization was to *hprt*, *gapdh*, and *β-actin*. qPCR data is presented using the ΔΔC(t) method [20].

### Primer Sequences for Real-time Quantitative PCR

Exon 1 forward: 5′ TGAGGCCTCTGAAGACCATG 3′.

Exon 2 reverse: 5′ TCAGCAGCTATAATGTCCATC 3′.

Exon 5 forward: 5′ GGGACATCCAACCTCAAAGA 3′.

Exon 6 reverse: 5′ CCGTAGTGGTGGAAATCTTG 3′.

5′ untranslated forward: 5′ GCGTCAAAGAGCCAATCAAC 3′.

Terminal exon 3 reverse: 5′ CTTGTCATTCTGATAGGCAGTA 3′.

### Generation of Anti-Tbx3 C-terminal Antibody

We synthesized a KLH conjugated peptide unique to the C-terminus of Tbx3: GLEAK PDRSCSGSP. The antiserum was generated in rabbits by Covance and the polyclonal antibody was affinity purified, and validated by western blot, immunoprecipitation and immunohistochemistry.

### Immunohistochemistry

E10.5 embryos were harvested in 1XPBS, fixed overnight at 4 degrees in 4% paraformaldehyde. Limb buds were dissected and processed for paraffin sectioning. Immunohistochemistry was carried out on 10 micron paraffin sections using Anti-Tbx3 C-terminal antibody. Citrate antigen retrieval was performed and sections incubated with primary antibody (1∶200) overnight at 4 degrees and detected using donkey anti-rabbit conjugated to Alexa fluor 488(1∶500) from Invitrogen. Nuclei were stained with Hoescht. Slides were imaged with a Nikon ARI inverted confocal microscope at the University of Utah Imaging Core.

### Statistical Analysis

Comparison of the recovered versus expected ratios of genotypes from mating male and female *Tbx3^+/Δ1-3^* and *Tbx3^+/Δflox^* heterozyogtes was done using Pearson’s chi-squared test. Comparison of the numbers of *Tbx3^+/Δ1-3^* and *Tbx3^+/Δflox^* adult females demonstrating abnormal pheontypes was done using Fisher’s exact test. Statistical test were performed using GraphPad software (www.graphpad.com).
